# Corticotectal Projections From the Premotor or Primary Motor Cortex After Cortical Lesion or Parkinsonian Symptoms in Adult Macaque Monkeys: A Pilot Tracing Study

**DOI:** 10.3389/fnana.2019.00050

**Published:** 2019-05-22

**Authors:** Michela Fregosi, Alessandro Contestabile, Simon Badoud, Simon Borgognon, Jérôme Cottet, Jean-François Brunet, Jocelyne Bloch, Martin E. Schwab, Eric M. Rouiller

**Affiliations:** ^1^Section of Medicine, Department of Neurosciences and Movement Sciences, Faculty of Science and Medicine, University of Fribourg, Fribourg, Switzerland; ^2^Fribourg Cognition Center, Fribourg, Switzerland; ^3^Platform of Translational Neurosciences, Fribourg, Switzerland; ^4^Swiss Primate Competence Center for Research (SPCCR), Fribourg, Switzerland; ^5^Cell Production Center (CPC), Lausanne University Hospital (CHUV), Lausanne, Switzerland; ^6^Department of Neurosurgery, Lausanne University Hospital (CHUV), Lausanne, Switzerland; ^7^Brain Research Institute, University of Zurich, Zurich, Switzerland

**Keywords:** non-human primate, anterograde tracing, motor cortex, brainstem, Parkinson, spinal cord injury, cortical lesion

## Abstract

The corticotectal projections, together with the corticobulbar (corticoreticular) projections, work in parallel with the corticospinal tract (CST) to influence motoneurons in the spinal cord both directly and indirectly *via* the brainstem descending pathways. The tectospinal tract (TST) originates in the deep layers of the superior colliculus. In the present study, we analyzed the corticotectal projections from two motor cortical areas, namely the premotor cortex (PM) and the primary motor cortex (M1) in eight macaque monkeys subjected to either a cortical lesion of the hand area in M1 (*n* = 4) or Parkinson’s disease-like symptoms PD (*n* = 4). A subgroup of monkeys with cortical lesion was subjected to anti-Nogo-A antibody treatment whereas all PD monkeys were transplanted with Autologous Neural Cell Ecosystems (ANCEs). The anterograde tracer BDA was used to label the axonal boutons both *en passant* and *terminaux* in the ipsilateral superior colliculus. Individual axonal boutons were charted in the different layers of the superior colliculus. In intact animals, we previously observed that corticotectal projections were denser when originating from PM than from M1. In the present M1 lesioned monkeys, as compared to intact ones the corticotectal projection originating from PM was decreased when treated with anti-Nogo-A antibody but not in untreated monkeys. In PD-like symptoms’ monkeys, on the other hand, there was no consistent change affecting the corticotectal projection as compared to intact monkeys. The present pilot study overall suggests that the corticotectal projection is less affected by M1 lesion or PD symptoms than the corticoreticular projection previously reported in the same animals.

## Introduction

In the central nervous system (CNS) of primates, there are several parallel descending projection systems originating from either the cerebral cortex or the brainstem. The cerebral cortex informs the spinal cord about the desired voluntary movements both directly *via* the corticospinal tract (CST) and/or indirectly *via* the corticorubral, corticotectal and the corticobulbar (corticoreticular) projections which connect the cerebral cortex with different levels of the brainstem that in turn projects to the spinal cord (Lemon, 2008).

Corticotectal projections originate in layer V of the cerebral cortex and act on the superior colliculus (SC; Fries, 1984, 1985). Motor cortical areas have been shown to send projections to the SC mainly to the intermediate and deep layers. The premotor area (PM), both dorsal (PMd) and ventral (PMv), as well as the supplementary motor area (SMA) project to the intermediate and deep layers of SC in intact monkeys (Fries, 1984, 1985; Borra et al., 2010, 2014; Distler and Hoffmann, 2015; Fregosi and Rouiller, 2017). Projections from M1 have also been found although less dense than those from PM and SMA (Fries, 1984, 1985; Tokuno et al., 1995; Fregosi and Rouiller, 2017).

The intermediate and deep layers of SC have been proposed to be a center of sensorimotor integration (Sparks and Hartwich-Young, 1989). These layers receive projections from the lateral grasping network (Borra et al., 2014), together with projections from motor cortical areas (Fries, 1984, 1985; Borra et al., 2010, 2014; Distler and Hoffmann, 2015; Fregosi and Rouiller, 2017), and are thus well placed to integrate visuomotor information of the object and action goal (Borra et al., 2014). Furthermore, the intermediate and deep layers of SC have been shown to possess neuronal populations that are related to reaching movement (Werner, 1993; Werner et al., 1997a,b) as well as to hand-object interaction (Nagy et al., 2006). Moreover, intracortical stimulation of SC has been shown to produce arm movements (Philipp and Hoffmann, 2014). Furthermore, from the intermediate and deep layers of SC originates the tectospinal tract (TST) that descends to the cervical upper spinal cord (Castiglioni et al., 1978; Nudo and Masterton, 1989; Nudo et al., 1993). Therefore, the presence of neurons related to reaching and hand movements approaching an object and also projections from various motor areas make the SC a likely player in movement control. Nevertheless, considering the specific motor control of finger movements (pure manual dexterity) in overtrained motor tasks (assimilated to motor habit: see Kaeser et al., 2013), requiring modest visuomotor integration due to over-practice, it is likely that the corticotectal and tectospinal projection systems are less crucial for manual dexterity than the corticoreticular and reticulospinal projection systems (Fregosi et al., 2017, 2018; Zaaimi et al., 2018). As a consequence, one may predict that the corticotectal projection from PM is less impacted after lesion in the hand representation of M1 than the corticoretricular projection (Fregosi et al., 2018). Similarly, the corticotectal projections from PM and M1 are likely less impacted in case of Parkinson’s disease-like symptoms (PD) than the corticoreticular projections (Fregosi et al., 2018), although there is evidence of pathophysiological changes in the system of control of saccades involving the frontal cortex and the SC in case of PD (Cubizolle et al., 2014; Terao, 2014).

Our goal was to investigate in eight lesioned adult macaque monkeys how corticotectal projections arising from PM and M1 are affected either by cortical lesion in M1 hand area or by Parkinson’s disease-like symptoms (PD). The present pilot tracing study has been conducted on the animals used to study corticobulbar projections from PM and M1 after different lesion/pathology and in presence/absence of treatment (Fregosi et al., 2018). The aim of the present analysis was to fill the gap on how ipsilateral corticotectal projections may rearrange, if they do, as well as their density and laminar distribution after M1 hand area cortical lesion or PD, with the hypothesis that the corticotectal projection (present study) is less impacted than the corticoreticular projection (Fregosi et al., 2018). The cases presented here are derived from previous research proposals initially aimed and specifically designed to address clinically relevant issues in non-human primates, some of them suitable for subsequent and complementary tracing analysis, with however a clear limitation related to the number of animals, as one may expect for monkey animal models.

## Materials and Methods

The materials and methods used in the present investigation are in all points similar to those already reported in recent publications related to the corticoreticular and corticotectal projections (Fregosi and Rouiller, 2017; Fregosi et al., 2017, 2018), and therefore are not repeated here in detail. Furthermore, the present data are derived from the same monkeys reported in a previous publication (Fregosi et al., 2018), with the exception that in the present investigation it was not possible to analyze spinal cord injury (SCI) monkeys for corticotectal projections as we did for corticobulbar projections due to the unavailability of the histological material at midbrain level. In particular, the methods used in the present study to analyze the histological sections are the same as those used to establish the corticotectal projections in intact monkeys (Fregosi and Rouiller, 2017).

In the present study (Table 1), eight macaque monkeys received unilateral BDA injections in either PM (*n* = 6) or M1 (*n* = 2) after being subjected to either unilateral cortical lesion of M1 hand area (*n* = 4; BDA injection in the adjacent intact PM) or PD (MPTP intoxication; *n* = 4; BDA injection in M1 in two monkeys and in PM in two monkeys). In five out of six monkeys injected in PM the BDA injection comprised both PMd and PMv, whereas for one monkey (MK-RO) the injection was restricted to PMd only. In the group of monkeys subjected to M1 lesion, BDA was injected in the adjacent ipsilesional intact PM, as the latter was found to contribute to the functional recovery (Liu and Rouiller, 1999; Hoogewoud et al., 2013). In the PD-like group, the effect of the intramuscular low-dose MPTP treatment was expected to be bilateral and therefore the unilateral BDA injection was performed in one hemisphere chosen randomly.

**Table 1:** Individual data for the eight monkeys included in the present study.

	Mk-MO	Mk-VA	Mk-RO	Mk-BI	Mk-LL	Mk-MY	Mk-LY	Mk-MI
BDA injection in	PMd/PMv	PMd/PMv	PMd	PMd/PMv	PMd/PMv	PMd/PMv	M1	M1
Age at sacrifice	6	6	4.5	6	7.5	9.5	7.5	9.5
Weight	5.6	4.9	3.2	5	3.6	4.3	3.3	3.3
Sex	Male	Male	Male	Male	Female	Female	Female	Female
Species	Fasc.	Fasc.	Fasc.	Fasc.	Fasc.	Fasc.	Fasc.	Fasc.
Type of lesion	MCI	MCI	MCI	MCI	MPTP	MPTP	MPTP	MPTP
Therapeutic treatment*	Nogo-A	Nogo-A	none	none	ANCE	ANCE	ANCE	ANCE
Nb. of series of sections	5	5	5	5	10	10	10	10
Intersections interval (μm)	250	250	250	250	500	500	500	500
Total BDA volume injected (μL)	10.8	5	4.8	7.2	9.7	11.5	9	9
Nb. of BDA injection sites	12	5	6	11	8	9	6	6
Body territory injected**	Large	Large	Large	Large	Large	Large	Large	Large
Volume lesion with ibotenic acid (mm^3^)	41.8	20	14	20.1	-	-	-	-
Loss DA neurons in SNpc (%)	-	-	-	-	67.4	71.8	38.8	73.4
Nb. labeled CS axons	1,975	1,312	543	1,328	593	611	1,671	1,117
Nb. boutons in SC	207	1,372	3,802	2,799	543	3,323	318	170
Nb. boutons in SCint	23	138	2,242	1,409	126	1,255	12	112
Nb. boutons in SCdeep	129	1,081	992	902	322	1,736	212	52
Corrected Nb. boutons in SC***	**207**	**1,372**	**3,802**	**2,799**	**1,086**	**6,646**	**636**	**340**
Normalized Nb. boutons in SC****	**105**	**1,046**	**7,002**	**2,108**	**1,831**	**10,877**	**381**	**304**

SC, Superior Colliculus; SCint, intermediate nucleus of SC; SCdeep, deep nucleus of SC. Fasc., macaca fascicularis. Type of lesion: MCI: motor cortex injury, corresponding to a unilateral infusion of ibotenic acid in the hand area of the primary motor cortex (M1), as previously reported (Liu and Rouiller, 1999; Kaeser et al., 2010; Hoogewoud et al., 2013; Wyss et al., 2013). MPTP: MPTP intoxication (intramuscular low-dose), as previously reported (Borgognon et al., 2017). *Two monkeys in the M1-lesion group were treated with an anti-Nogo-A antibody, as previously reported (Wyss et al., 2013). The PD-like monkeys were all treated with the ANCE cellular therapy, as previously reported (Bloch et al., 2014; Borgognon et al., 2017). **In both PM and M1, the BDA injections covered most of the targeted areas (see Fregosi et al., 2017) and were not preceded by ICMS (intracortical microstimulation) sessions. ***In each monkey, the number of axonal boutons in SC was corrected to take into account the differences in intersections interval (7th row from top), as explained in the “Materials and Methods” section (no corrections for the four monkeys with the injections in PM and five series taken as reference). ****The corrected number of axonal boutons in SC (line above) was finally normalized according to the number of corticospinal BDA labeled axons, as explained in the “Materials and Methods” section. Bold values indicates the most pertinent values.

Typically, the post-lesion functional recovery period (day of lesion to day of euthanasia) is several months (6 months or more). The BDA injections took place usually about 30 days before euthanasia. In other words, BDA was injected in an intact cortical area at a time point when the functional recovery (most often incomplete) has already taken place, when the circuits have been re-organized and thus can be considered stable. After such post-lesion long delay to inject BDA, the concern that the lesion surgery may influence the tracer uptake is not relevant.

The injection sites of BDA are the same as those reported in Fregosi et al., 2018 (their Figure 1). Furthermore (Table 1), six out of eight monkeys were subjected to post-lesion treatment: two monkeys with cortical lesion of M1 hand area were treated with the anti-Nogo-A antibody, whereas PD monkeys were subjected to the autologous neural cell ecosystem (ANCE); treatment protocols are the same as those reported recently (Fregosi et al., 2018; see also Wyss et al., 2013; Borgognon et al., 2017). Two monkeys subjected to M1 lesion were not treated (Table 1).

As a result of BDA injection in M1 or PM, anterogradely labeled axonal branches were found in the ipsilateral SC, forming spatially restricted axonal terminal fields exhibiting boutons *en passant or terminaux*. As previously reported (Fregosi et al., 2017), a bouton is defined as a swelling of a diameter of at least twice the diameter of the attached axonal branch. All boutons visible in SC were plotted on the analyzed histological sections (see below “exhaustive plotting” method), without however counting separately boutons *en passant* and boutons *terminaux*. The distinction between the two types of boutons is not 100% accurate: for instance, in the case of a bouton *terminal* identified as such on a histological section, it may happen that the axonal branch continues on the adjacent section (which is not available in case the adjacent series of sections has been used for another marker). Nevertheless, as previously reported for corticobulbar and corticotectal projections in intact monkeys (Fregosi and Rouiller, 2017; Fregosi et al., 2017), boutons *en passant* are far more numerous than boutons *terminaux*.

**Figure 1:**
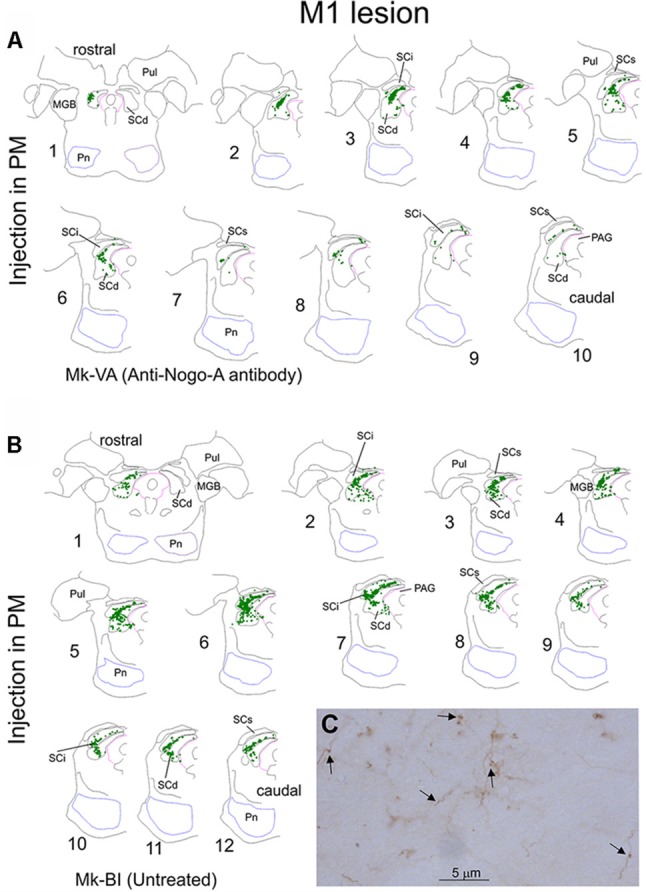
Typical distribution of BDA labeled corticotectal axonal boutons in the ipsilateral superior colliculus (SC) in two representative monkeys subjected to unilateral primary motor cortex (M1) lesion **(A)**. In both monkeys, BDA was injected in the ipsilesional premotor cortex (PM). In panel **(A)**, Mk-VA was treated with an anti-Nogo-A antibody whereas in Mk-BI was untreated **(B)**. Only the ipsilesional SC is shown. Axonal boutons are depicted with green dots. The histological sections are arranged from rostral to caudal. The **(C)** illustrates a typical BDA labeled terminal field in the SC, with axon segments as well as a few boutons pointed by arrows. See list of abbreviations.

**Figure 2:**
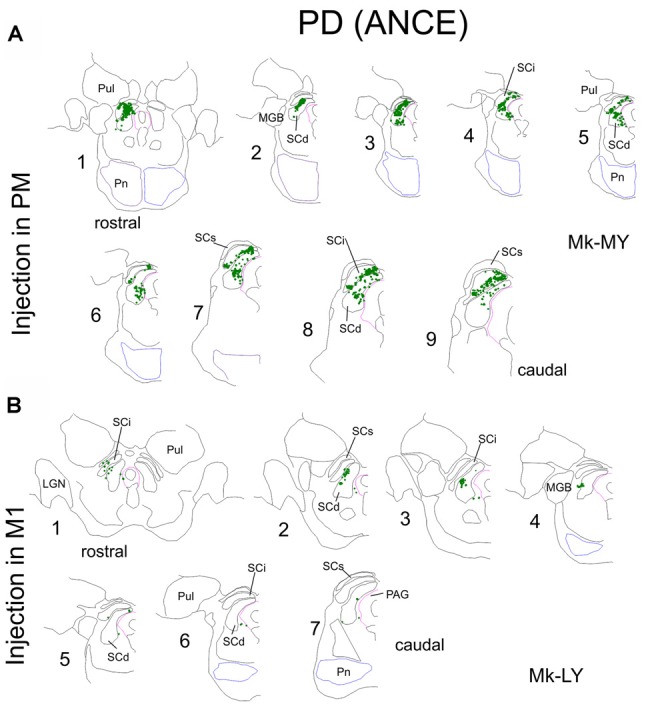
Same as in Figure 1, but for two representative Parkinson’s disease (PD) monkeys. Note that one monkey (Mk-MY, **A**) was injected with BDA in PM, whereas the injection was in M1 in the second monkey (Mk-LY, **B**).

All monkeys were previously involved in behavioral tasks (Kaeser et al., 2010, 2011, 2014; Schmidlin et al., 2011; Bashir et al., 2012; Hamadjida et al., 2012; Hoogewoud et al., 2013; Wyss et al., 2013; Badoud et al., 2017; Borgognon et al., 2017). All surgical experimental procedures, experiments and animal care were conducted in respect to the ethical guidelines (ISBN 0-309-05377-3, 1996) and authorized by the local (Canton of Fribourg) and federal (Switzerland) veterinary authorities (veterinary authorization numbers FR156-04, FR156-06, FR-185-08, FR-17-09, FR-2012-01, FR-2012-01E). All procedures for anesthesia, surgery, treatments as well as euthanasia are the same as those reported earlier (Wannier et al., 2005; Freund et al., 2006, 2007; Schmidlin et al., 2011; Wyss et al., 2013; Borgognon et al., 2017). Histological preparation of the tissue is the same as that recently reported (Fregosi and Rouiller, 2017; Fregosi et al., 2017, 2018). As for intact animals (Fregosi and Rouiller, 2017), the present analysis was restricted to the ipsilateral SC with respect to the tracer injection (Table 1) and was performed according to the same criteria as previously reported (Fregosi and Rouiller, 2017). Using the software Neurolucida (MBF, Bioscience-MicroBrightField, Inc. Version 11), the BDA labeled axonal boutons (both *terminal* and *en passant*) were charted at a total magnification of 200× (objective of 20×, no oil immersion used; Figures 1, 2). At that total magnification, the focal plane did not cover the entire depth of the histological section (50 μm), thus requesting to continuously adjust the *z* axis at each consecutive scanned window. As illustrated in Figure 1, the BDA labeled axonal terminal fields were spatially restricted and moderately dense, allowing an exhaustive plotting of all axonal boutons in the superior colliculus, instead of stereological sampling (see Fregosi and Rouiller, 2017). Furthermore, the subdivision of the SC in layers was performed according to the Paxinos atlas (Paxinos et al., 2000).

In order to allow a direct comparison of corticotectal projections between monkeys due to the difference in BDA injection size and volume the data were normalized according to the number of BDA-labeled CS axons calculated just above the pyramidal decussation (Table 1). Moreover, the midbrain was cut at 50 μm in a variable number of series across animals (5 or 10, see Table 1). To avoid under-quantification due to the distance between the analyzed sections we corrected the data as was previously done for intact animals (Fregosi and Rouiller, 2017). Here, we took as reference sectioning in five series (cortical lesion) as we did for intact animals injected in PM. Brain sections of PD animals were collected in 10 series and therefore the normalized and corrected numbers of boutons were multiplied by a factor of 2. In Mk-RO, five histological sections located in the middle of SC were not available and thus were not quantified. BDA injections in M1 were not precisely located on a body region in particular although including the hand area.

## Results

The two groups of M1-lesion or PD-like monkeys were derived from previous studies in which the behavioral data were reported previously in detail (M1 lesion: Hoogewoud et al., 2013; Wyss et al., 2013; PD-like monkeys: Borgognon et al., 2017). These behavioral properties are not repeated in detail in the present article, focused on the corticotectal projection. Briefly, in M1 lesioned monkeys, the anti-Nogo-A antibody treatment enhanced the functional recovery of manual dexterity, as compared to untreated monkeys (Hamadjida et al., 2012; Hoogewoud et al., 2013; Wyss et al., 2013). Furthermore, the callosal projection from the intact hemisphere to the premotor cortex (PM) adjacent to the M1 lesion was increased in anti-Nogo-A antibody treated monkeys, as compared to untreated monkeys (Hamadjida et al., 2012). Finally, the corticobulbar (corticoreticular) projection originating from PM adjacent to the M1 lesion was reduced as compared to intact monkeys, but without difference between anti-Nogo-A antibody treated monkeys and untreated monkeys (Fregosi et al., 2018). These various changes in connectivity after the M1 lesion may have contributed (directly or indirectly) to the functional recovery, either spontaneous (untreated monkeys) and/or the recovery enhanced by the treatment (anti-Nogo-A antibody).

In monkeys with Parkinson symptoms (PD), the ANCE treatment enhanced the functional recovery of global motor abilities (clinical score, locomotion; see Borgognon et al., 2017), as well as manual dexterity (Borgognon et al., 2019). In these ANCE treated PD monkeys, the corticobulbar projection was also reduced as compared to intact monkeys, though more prominently for the projection originating from PM than from M1. Both treatments (anti-Nogo-A antibody and ANCE) do not affect the general behavior and health of the monkeys, as reported earlier (e.g., Freund et al., 2006, 2009; Kaeser et al., 2011; Hamadjida et al., 2012; Wyss et al., 2013; Bloch et al., 2014; Badoud et al., 2017; Borgognon et al., 2017, 2019).

As observed in intact monkeys (Fregosi and Rouiller, 2017), the corticotectal projection from M1 and PM in the eight monkeys of the present study is massively ipsilateral, with very sparse if any projections to the opposite superior colliculus. For this reason, the present analysis was limited to the ipsilateral superior colliculus with respect to the injected motor cortical area.

### Corticotectal Projections to SC From PM in Monkeys With Lesion of M1 Hand Area

The anterograde tracer BDA was injected in both PMd and PMv in Mk-MO, Mk-VA and Mk-BI, whereas MK-RO was injected in PMd only (see Fregosi et al., 2018, their Figure 1 for a representation of the injection sites). Mk-MO and MK-VA were treated with anti-Nogo-A antibody post-lesion whereas both Mk-RO and Mk-BI remained untreated. Since all animals had five series of brain sections, which has been used as reference, no correction was necessary with respect to the intersection intervals (Table 1).

Figure 1 shows the representative distribution of corticotectal axonal boutons in the SC ipsilateral to the BDA injection site in PM in one monkey treated with anti-nogo-A antibody (Figure 1A) and in one monkey without treatment (Figure 1B). Projections were located in the intermediate (SCint) and deep (SCdeep) SC layers in both monkeys throughout the entire SC rostrocaudal extent. Mk-BI exhibited a stronger corticotectal projection than Mk-VA (Figure 1). Furthermore, in Mk-BI the boutons were found in both ventro-lateral and dorso-medial sectors of the SC with a majority of boutons in its ventro-lateral part, whereas in Mk-VA there is no clear preponderance for ventro-lateral or dorso-lateral part of SC.

**Figure 3:**
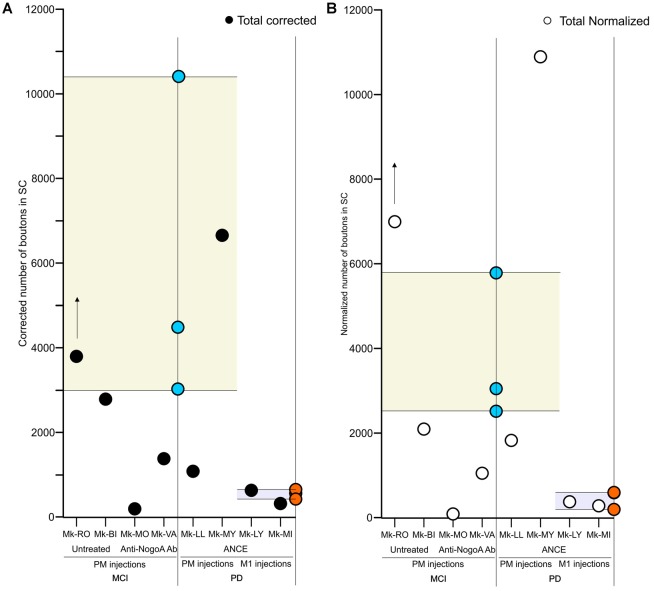
Scatter plots of the total numbers of corticotectal boutons observed in the SC in the different groups of monkeys subjected to motor cortex injury (MCI) or MPTP intoxication (PD). The data are restricted to the ipsilateral SC with respect to the BDA injection site, either in PM or M1. For comparison, the corresponding data in intact monkeys (Fregosi and Rouiller, 2017) are represented here by the range derived from intact cases (yellow or light blue areas), with individual data points in blue (PM projection in intact monkeys) or in brown (M1 projection in intact monkeys). The individual data points for the corticotectal projections (present study) are indicated with black or open white symbols for absolute data or normalized data, respectively (**A,B**, respectively). The BDA injection site (PM or M1) is indicated below the graph. Panel **(A)** is for the absolute numbers of corticotectal boutons, whereas **(B)** is for normalized numbers of corticotectal boutons. The presence/absence of treatment is indicated below the graphs. In both panels, the data were corrected with respect to the distance between consecutive sections (see “Materials and Methods” section). For Mk-RO, the vertical arrow indicates that the number of axonal boutons in SC was underestimated, due to a few missing histological sections (see “Results” section).

First, we compared in SC the amount of axonal boutons after BDA injections in PM in monkeys (*n* = 4) subjected to a unilateral cortical lesion of the M1 hand area and in intact animals (*n* = 3, see Fregosi and Rouiller, 2017 for corticotectal projections in intact animals). Mk-MO and Mk-VA (anti-Nogo-A antibody treated) showed a decreased corticotectal projection as compared to intact animals, with the strongest decrease in Mk-MO, considering both the absolute numbers of boutons (Figure 3A) and the normalized numbers of boutons (Figure 3B). The effect of the M1 lesion is different in the two untreated monkeys. In Mk-BI (untreated), the numbers of boutons are close to the inferior limit of the range observed in intact monkeys, irrespective of normalization of the data or not (Figures 3A,B). In Mk-RO (untreated), the results are inconsistent whether considering the absolute vs. the normalized numbers of boutons, although they remain fairly close to the range observed in intact monkeys (Figures 3A,B). However, five histological sections of SC in Mk-RO were unavailable. It is thus possible that, if these missing sections would have been included, the amount of boutons would have been higher than reported in Figures 3A,B.

**Figure 4:**
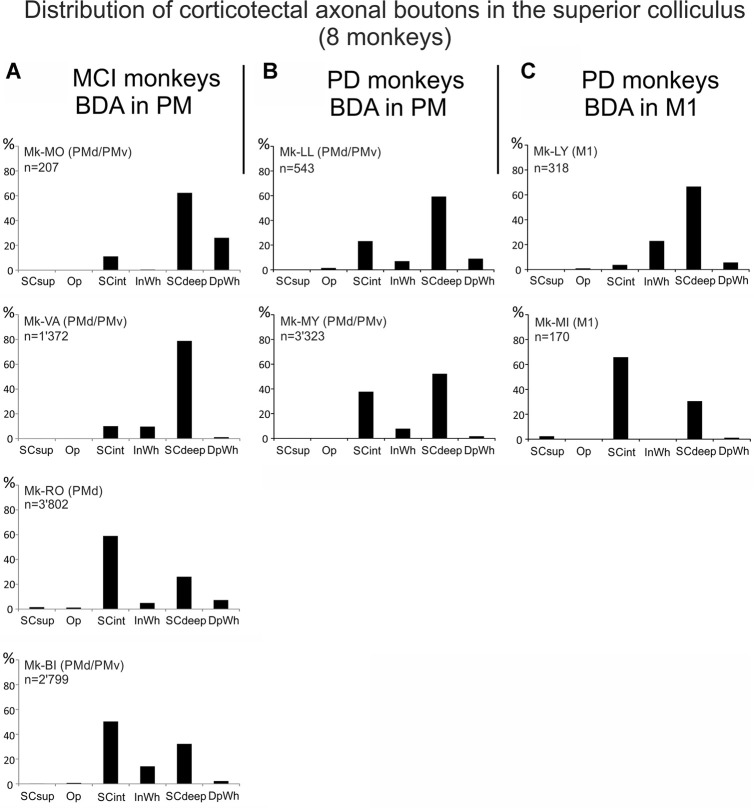
Distributions of the numbers of BDA-labeled corticotectal axonal boutons both *en passant* and *terminaux* in the ipsilateral SC, across the different SC layers in each monkey (see “List of Abbreviations”), subjected to cortical lesion of the hand area in M1 motor cortex injury (MCI, **A**), or to MPTP intoxication (PD, **B,C**). In **(A)**, the top two monkeys were treated with the anti-Nogo-A antibody, whereas the bottom two monkeys were untreated. In panels **(B,C)**, all monkeys were autologous neural cell ecosystems (ANCEs) treated. In each graph, the sum of all bins is 100%.

We further analyzed the distribution of corticotectal axonal boutons in the SC layers (Figure 4A). In the two monkeys treated with anti-Nogo-A antibody, both with BDA injection in both PMd and PMv, the large majority of boutons were located in SCdeep whereas a small percentage of them was found in SCint. In contrast, in Mk-RO and Mk-BI (untreated animals), the boutons were more equally distributed between SCint and SCdeep, although SCint was predominant in both monkeys (Figure 4A). Absent or only very sparse corticotectal boutons were found in the superficial layer of SC (Figure 4A). There were not enough cases in order to tentatively correlate the number of boutons with the size of the M1 lesion, especially considering the further subgrouping based on the presence/absence of anti-Nogo-A antibody treatment.

### Corticotectal Projections to SC From PM and M1 in PD Monkeys

In PD animals the tracer BDA was injected in both PMd and PMv in Mk-LL and Mk-MY, whereas it was injected in M1 in Mk-LY and Mk-MI. All four PD monkeys were treated with ANCE (see “Materials and Methods” section). The distribution of BDA labeled corticotectal boutons in SC is illustrated in two representative PD monkeys (Figure 2), one injected in PM (Mk-MY) and one injected in M1 (Mk-LY). Since all PD animals had 10 series of brain sections, the numbers of boutons were further corrected (multiplied) by a factor of 2.

As in intact animals (Fregosi and Rouiller, 2017), the corticotectal projections in the PD monkeys were stronger when originating from PM than from M1 (Figures 2, 3A,B). Furthermore, there is no clear predominance in either the ventro-lateral or medio-dorsal parts of SC: corticotectal boutons are located in both SCint and SCdeep with a majority of corticotectal projections to SCdeep (Figure 2). Corticotectal boutons were found in the rostral part of SC but not in the caudal-most sections (Figure 2). As compared to the intact monkeys (Figures 3A,B; *n* = 3 for each injected area PM or M1), the number of corticotectal boutons in SC after BDA injection in M1 in the 2 PD monkeys is similar to those found in the intact monkeys. In the two PD monkeys injected in PM, the number of corticotectal boutons in SC was lower in Mk-LL than in intact monkeys, whereas this was different in Mk-MY (comparable for absolute numbers but higher for normalized numbers, with respect to intact monkeys).

The corticotectal boutons in both PM and M1 injected animals were found in SCint and SCdeep layers of SC (Figures 4B,C). In both Mk-LL and Mk-MY (PM injection) the majority of boutons was found in SCdeep. The same was true for Mk-LY (M1 injection) which in turn showed a very sparse projection to SCint. On the contrary, Mk-MI showed a denser corticotectal projection to SCint as compared to SCdeep. Absent or only sparse projections were found to the superficial SC layers. Again, as for M1 lesion cases, the PD cases were not numerous enough to tentatively correlate the numbers of corticotectal boutons in SC in the four PD monkeys with the percent loss of dopaminergic neurons in the substantia nigra pars compacta, as reported earlier (Borgognon et al., 2017), especially considering the subgrouping with respect to the site of BDA injections (PM vs. M1).

## Discussion

Our aim in this pilot analysis was to tentatively investigate on a limited number of monkeys whether and how corticotectal projections may rearrange following a lesion (M1) or a pathology (PD) affecting the CNS. To the best of our knowledge, this is the first pilot study assessing the possible rearrangement of corticotectal projections in non-human primates after lesion or pathology of the CNS. Although limited to a restricted number of monkeys (see below), the data suggest that the corticotectal projections from PM tend to rearrange (decrease) following M1 hand area cortical lesion and subsequent anti-Nogo-A antibody treatment; this is not the case when the M1 lesion was not followed by a treatment (Figure 3). In PD monkeys the corticotectal projections from M1 and PM did not tend to substantially change their density as compared to intact animals (Figure 3).

In spite of a low number of monkeys, our recent study (Fregosi et al., 2018) showed substantial changes of the corticobulbar (corticoreticular) projections originating from PM and M1 after unilateral lesion of M1 or PD. The corticotectal projection was investigated here on the very same (limited) cohort of monkeys, with the hypothesis that fewer changes after M1 lesion or PD are expected on the corticotectal projection, as compared to the corticobulbar projection. The results tend to support this hypothesis, as the corticotectal projection was moderately affected (decreased) after M1 lesion (only when anti-Nogo-A antibody treated), whereas there was no consistent change in PD monkeys with ANCE treatment. As the cohort of monkeys is the same in both studies, the fairly strong difference between the two projection systems (corticobulbar vs. corticotectal) is suggestive of a putative distinct role played by these 2 projection systems in the functional recovery, with however the residual doubt due to the low number of cases.

Corticotectal projections to the SC are directed mainly to the intermediate and deep layers (Figure 3) as in intact animals (Fries, 1984, 1985; Distler and Hoffmann, 2015). However, there was a difference in the distribution across the SC layers between treated and untreated animals injected with BDA in PM and subjected to M1 lesion: in monkeys injected in both PMd and PMv and receiving a treatment (anti-Nogo-A antibody), the majority of boutons were found in SCdeep whereas in untreated monkeys the majority of boutons were located in SCint (Figure 4). Projections to SC from M1 ended in PD monkeys generally in the deep layers (Figure 4), except in one monkey (Mk-MI).

### Limitations

The present study involves a limited number of monkeys (*n* = 8) subjected either to cortical lesion (*n* = 4) or to pathology (PD; *n* = 4), as one may reasonably expect from a non-human primate study, mostly for ethical reasons. Furthermore, in each group of monkeys there was a further subdivision in two subgroups: for the cortical lesion (*n* = 4) only two monkeys received the anti-Nogo-A antibody treatment whereas two monkeys remained untreated; for PD monkeys (*n* = 4; all treated with ANCE) two monkeys were injected with BDA in PM and two animals in M1. Thus, each subgroup was composed of two monkeys only.

Furthermore, as there was no PD monkey without the ANCE treatment, the information on how the corticotectal projections would have evolved in PD untreated monkeys is still missing. Chronologically, in order to demonstrate the beneficial effect of ANCE, two fairly large groups of St-Kitts monkeys with PD were compared, one with ANCE treatment and the other one without treatment (Bloch et al., 2014). Unfortunately, no BDA injection was performed in those monkeys at that time, preventing the analysis of connectivity at that early stage. As the proof of principle for ANCE was thus verified (Bloch et al., 2014), the second (present) step was to specifically investigate aspects not covered at the first step, such as dopaminergic activity in the striatum measured with PET (Borgognon et al., 2017) and the functional recovery of manual dexterity (Borgognon et al., 2019). In this second step conducted in Switzerland, with very strict ethical guidelines to restrict drastically the number of monkeys used for research, the protocol was limited to four monkeys, all treated with ANCE. Here, the strategy was to compare intra-individually the PET and behavioral parameters at two time points, namely post MPTP intoxication and post ANCE implantation (Borgognon et al., 2017, 2019). This explains why there is no PD monkey without ANCE available with BDA injection to establish the corticotectal projection in the absence of treatment.

In addition, in cortical lesioned monkeys (M1) as well as in PD monkeys, corticotectal projections from SMA still need investigation. SMA has been shown to be involved in the functional recovery after large cortical lesions (McNeal et al., 2010). We could thus speculate that its corticotectal projections, which we demonstrated to be as strong as those from PM in intact animals (see Fregosi and Rouiller, 2017), may have played a role in the functional recovery.

A further limitation of this study is the time point of the anatomical analysis. The data show the plastic changes at about 3–8 months post-lesion when the monkey reached a post-lesion plateau of performance (for monkeys with cortical lesion see Kaeser et al., 2011; Wyss et al., 2013). Thus, we cannot exclude that during the immediate (early) recovery phase following the lesion there was a different density pattern of corticotectal projections than that obtained in the present study. This suggestion is linked with the fact that it has been shown in rodents that after a lesion there is first sprouting and then subsequent pruning of the projections (for review, see Pernet and Schwab, 2012). This suggests that different patterns might be found at different time points during the post-lesion period.

Finally, the pros and cons of the normalization procedure of the data (see Figure 3 and as explained in the “Materials and Methods” section) have been discussed in detail in recent publications (Fregosi and Rouiller, 2017; Fregosi et al., 2017, 2018) and are therefore not mentioned here any further. In any case, both the absolute and normalized data are provided here (Figure 3 and Table 1), allowing comparison between them and freedom to give more emphasis to one or the other.

### M1 Cortical Lesion Changes the Corticotectal Projection From PM in Anti-Nogo-A Antibody Treated Monkeys

We observed a decrease of the corticotectal projections from PM in monkeys subjected to M1 lesion and treated with anti-Nogo-A antibody, but not in untreated animals (Figure 3). As recently reported (Fregosi et al., 2017), the corticobulbar projections from PM in cortical lesioned monkeys (M1) were strongly decreased but, in this case, both in presence or absence of the anti-Nogo-A antibody treatment. In other words, in anti-Nogo-A antibody treated monkeys, both corticobulbar and corticotectal projections from PM decreased after M1 lesion. In contrast, in the two untreated monkeys, the corticotectal projection from PM did not change whereas the corticobulbar projection was decreased (Fregosi et al., 2018). As far as the corticotectal projection is concerned (Figure 3), the untreated Mk-RO actually exhibited an increase of its corticobulbar projection when data were normalized, which was not the case in the other untreated monkey (Mk-BI), which did not change. At that step, this special observation in Mk-RO has to be put in perspective that this animal represents some sort of outlier: first, the M1 lesion was small (Table 1; see also Wyss et al., 2013; Contestabile et al., 2018) and moreover its lesion was performed in several steps (infusion of ibotenic acid at multiple steps) in contrast to the other M1 lesioned monkeys in which ibotenic acid was injected at once.

When comparing the corticotectal and corticobulbar projections, as a result of M1 lesion and anti-Nogo-A antibody treatment both projections were modified in the same direction as expected, namely a decrease of the density of these two corticofugal projections as compared to intact monkeys. The net result would then be that after M1 lesion and treatment, both the reticulospinal and tectospinal projections would become more independent from motor cortical areas, a condition which may be favorable for the enhancement of functional recovery observed in the treated monkeys (Hamadjida et al., 2012; Wyss et al., 2013). Surprising is the discrepancy in M1 lesioned monkeys and untreated: no change of corticotectal projection (Figure 3) but decrease of the corticobulbar projection (Fregosi et al., 2018). More monkeys would be needed to assess whether this difference is related to different mechanisms of spontaneous functional recovery in the absence of treatment.

The anti-Nogo-A antibody treatment is primarily expected to enhance axonal sprouting following a lesion, by making the CNS environment permissive for regeneration (see e.g., Pernet and Schwab, 2012; see also Freund et al., 2007: sprouting of corticospinal axons after cervical cord hemisection). In a global mechanism underlying functional recovery, one cannot exclude that some projection systems may be enhanced (sprouting), while some others may be reduced, in order to guarantee a coherence of the overall adaptation taking place in the multiple surviving neural systems. The decrease of the corticobulbar projection (Fregosi et al., 2018) and corticotectal projection, though to a much lesser extent (present study), may parallel enhancement of other projection systems, for instance the corticospinal projection, the corticocortical projections (Dancause et al., 2005), the reticulospinal projection, the rubrospinal projection, the callosal connectivity and many others. Ideally, to have a global and comprehensive picture, it would be necessary to be able to study all projections systems at the same time in the same animal following a specific lesion or pathology, in order to infer the complexity and flexibility of the multiple mechanisms underlying functional recovery.

### Corticotectal Projections From PM or M1 in PD Monkeys and in Presence of ANCE Treatment

As shown in Figure 3, one is tempted to conclude that the corticotectal projection in PD monkeys and treated with ANCE was not modified when originating from M1 and most likely also from PM. In the latter case, the situation is a bit less clear as one animal (Mk-LL) rather showed a moderate decrease of density of corticotectal projection as compared to intact animals whereas the other monkey showed an increase (Mk-MY). The latter observation in Mk-MY cannot be explained neither by a particular extent of DA neurons loss in the substantia nigra nor by a special degree of functional recovery from the MPTP lesion (Borgognon et al., 2017). It may be considered that the corticotectal projection from PM may be strongly variable from one animal to the next, as this actually appears in the group of the three intact monkeys (Figure 3; Fregosi and Rouiller, 2017: Mk-R13 with a clearly denser corticotectal projection than the other two intact monkeys).

The laminar distribution of corticotectal boutons in SC originating from PM in Mk-LL and Mk-MY was similar to that found in intact animals, with projections mainly in SCdeep (Figure 4). In contrast, the laminar distribution was distinct in the two monkeys injected with BDA in M1: mainly in SCdeep in Mk-LY and mainly in SCint in Mk-MI. Notice that these two monkeys are quite different in terms of the extent of DA neurons loss (Table 1; see also Borgognon et al., 2017) and furthermore Mk-MI was functionally much more affected by the MPTP lesion than Mk-LY. Whether this change of laminar distribution in SC in Mk-MI is related to specific mechanisms of functional recovery in case of severe PD symptoms remains speculative at that step, although plausible.

### Functional Meaning

The SC contains reach-related as well as hand-related neurons (Werner, 1993; Werner et al., 1997a,b; Nagy et al., 2006), it elicits arm movements when electrically stimulated (Philipp and Hoffmann, 2014) and it is also a site for visuomotor/sensorimotor integration (Borra et al., 2014). These influences can be sent to the upper cervical spinal cord *via* the TST, which originates from the intermediate and deep layers of SC (Castiglioni et al., 1978; Nudo et al., 1993), with in addition an influence from the motor cortical areas *via* the corticotectal projections. In addition, the tectospinal projections from the SC and corticospinal projections from PMd to the cervical spinal cord terminate in the same regions of the ventral horn, indicating that the SC may have a role in the modulation of arm and head movements (Distler and Hoffmann, 2015).

In line with our hypothesis, the present changes of corticotectal projections observed after M1 lesion or PD are less prominent than the changes observed for the corticobulbar projections (Fregosi et al., 2018). In case of PD, the corticotectal projection was largely unaffected (present study), whereas the corticobulbar projection was reduced, especially when originating from PM (Fregosi et al., 2018). After M1 lesion, both the corticoreticular and corticotectal projections were reduced, but more dramatically for the corticoreticular projection (Fregosi et al., 2018) than the corticotectal projection, change limited in the latter in anti-Nogo-A antibody treated monkeys (not in untreated monkeys). Form this comparison, it can be tentatively concluded that the corticoreticular projection plays a more important role in the functional recovery from motor disorders such as M1 lesion or PD than the corticotectal projection. This conclusion is consistent with the very significant role played by the reticular formation in the bilateral control of fractionated movements in intact monkeys (Zaaimi et al., 2018), more than the cortico-tecto-spinal system playing a less prominent role in that context. Indeed, the cortico-tecto-spinal system is largely unilateral (Castiglioni et al., 1978; Fries, 1984, 1985; Nudo and Masterton, 1989; Nudo et al., 1993; Borra et al., 2010, 2014; Distler and Hoffmann, 2015; Fregosi and Rouiller, 2017). Moreover, on the evolutionary point of view, the tectospinal projection system was found to be quite limited in size in primates and therefore does not belong to the major descending tracts (Nudo and Masterton, 1989).

A reduction of the corticobulbar (massive) and corticotectal projection (modest to moderate) after M1 lesion or PD may be interpreted as an adaptation mechanism, possibly related to functional recovery, by which the descending projections from the brainstem (reticulospinal projection) and from the tectum (tectospinal projection) to the spinal cord become more independent from motor cortical influences. More autonomy of these subcortical projections systems to the spinal cord may represent a contribution to the functional recovery, in combination with changes taking place in other surviving neural circuits (cortical level, corticospinal projection, basal ganglia, etc.). The actual contribution of a change in connectivity to functional recovery cannot be directly proven, at least at the present stage in this model. In the future, selective and reversible inactivation tools may permit to address this issue.

Overall, based on the two studies (Fregosi et al., 2018 and the current one), there is preliminary evidence that the corticobulbar projection may be more subjected to rearrangement post-lesion of M1 or PD-like symptoms, possibly in relation to the mechanisms of functional recovery, than the corticotectal projection. Ideally, a specifically designed further and more extensive tracing study would be needed in order to confirm this preliminary conclusion, involving larger cohorts of monkeys. However, such a proposal may not be realistic considering the most recent ethical concerns, recommending for good reasons a reasonable, responsible and limited use of non-human primates in biomedical research.

## Ethics Statement

All surgical experimental procedures, experiments and animal care were conducted in respect to the ethical guidelines (ISBN 0-309-05377-3, 1996). The study was reviewed and approved by the ethical committee of the Canton of Fribourg (“Commission de surveillance de l’expérimentation animale”) and authorized by the local (Canton of Fribourg) and federal (Switzerland) veterinary authorities (veterinary authorization numbers FR156-04, FR156-06, FR-185-08, FR-17-09, FR-2012-01, FR-2012-01E).

## Author Contributions

ER and MF designed the tracing analysis and drafted the manuscript. MF, AC and ER analyzed the histological sections. SBa, SBo, JC, J-FB, JB and ER designed and performed the MPTP experiments. ER and MS designed the anti-Nogo-A antibody treatments.

## Conflict of Interest Statement

The anti-Nogo-A antibody was provided by Novartis AG. The authors declare that the research was conducted in the absence of any commercial or financial relationships that could be construed as a potential conflict of interest.

## Abbreviations

BDA: biotinylated dextran amine
DpWh: deep white layer of SC
InWh: intermediate white layer of SC
M1: Primary motor cortex
MGB: medial geniculate body
OP: optic nerve layer of SC
PM: Premotor cortex
PMRF: Ponto-Medullary Reticular Formation
PMv: ventral premotor cortex
PMd: dorsal premotor cortex
Pn: pontine nuclei
Pul: pulvinar nucleus of the thalamus
SC: superior colliculus
SCsup: superior layer of SC
SCint: intermediate layer of SC
SCdeep: deep layer of SC
SMA: supplementary motor area
SMA-proper: caudal part of SMA
pre-SMA: rostral part of SMA.
